# Intracytoplasmic Sperm Injection Using DNA-Fragmented Sperm in Mice Negatively Affects Embryo-Derived Embryonic Stem Cells, Reduces the Fertility of Male Offspring and Induces Heritable Changes in Epialleles

**DOI:** 10.1371/journal.pone.0095625

**Published:** 2014-04-17

**Authors:** Priscila Ramos-Ibeas, Alexandra Calle, Raúl Fernández-González, Ricardo Laguna-Barraza, Eva Pericuesta, Antonia Calero, Miguel Ángel Ramírez, Alfonso Gutiérrez-Adán

**Affiliations:** Dpto. de Reproducción Animal y Conservación de Recursos Zoogenéticos, Instituto Nacional de Investigación y Tecnología Agraria y Alimentaria, Madrid, Spain; Rutgers University -New Jersey Medical School, United States of America

## Abstract

Intracytoplasmic sperm injection (ICSI) in mice using DNA-fragmented sperm (DFS) has been linked to an increased risk of genetic and epigenetic abnormalities both in embryos and offspring. This study examines: whether embryonic stem cells (ESCs) derived from DFS-ICSI embryos reflect the abnormalities observed in the DFS-ICSI progeny; the effect of DFS-ICSI on male fertility; and whether DFS-ICSI induces epigenetic changes that lead to a modified heritable phenotype. DFS-ICSI-produced embryos showed a low potential to generate ESC lines. However, these lines had normal karyotype accompanied by early gene expression alterations, though a normal expression pattern was observed after several passages. The fertility of males in the DFS-ICSI and control groups was compared by mating test. Sperm quantity, vaginal plug and pregnancy rates were significantly lower for the DFS-ICSI-produced males compared to *in vivo*-produced mice, while the number of females showing resorptions was higher. The epigenetic effects of DFS-ICSI were assessed by analyzing the phenotype rendered by the *Axin1^Fu^* allele, a locus that is highly sensitive to epigenetic perturbations. Oocytes were injected with spermatozoa from *Axin1^Fu^* ^/+^ mice and the DFS-ICSI-generated embryos were transferred to females. A significantly higher proportion of pups expressed the active kinky-tail epiallele in the DFS-ICSI group than the controls. In conclusion: 1) ESCs cannot be used as a model of DFS-ICSI; 2) DFS-ICSI reduces sperm production and fertility in the male progeny; and 3) DFS-ICSI affects the postnatal expression of a defined epigenetically sensitive allele and this modification may be inherited across generations.

## Introduction

The *in vitro* fertilization procedure intracytoplasmic sperm injection (ICSI) is currently the most commonly used method to overcome male infertility. However, concern has been recently raised that ICSI bypasses the natural selection process of the fertilizing sperm [1], allowing sperm with fragmented or otherwise damaged DNA to fertilize an oocyte [2]. Sperm DNA integrity is crucial for paternal reproductive potential and many studies have shown that the sperm of infertile men have more DNA strand breaks or other types of DNA damage than the sperm of fertile donors [3], [4]. We recently reported that ICSI using DNA-fragmented sperm (DFS) gives rise to genetic and epigenetic alterations in preimplantation embryos. These modifications include delayed male pronucleus demethylation, different sized telomeres, altered gene expression at the blastocyst stage, and modified expression of imprinting genes [5]. However, some of these suboptimal blastocysts are capable of implantation and our data suggest that the use of DFS for ICSI can produce effects later on in life such as aberrant growth, premature ageing, abnormal behavior, and mesenchymal tumors [5]. However, it is not yet known if ICSI may affect the fertility of the adult male offspring.

Embryonic stem cells (ESC) are clonal populations of cultured cells derived from the blastocyst-stage embryo that can give rise to all of the cell types that constitute the adult organism, and offer an *in vitro* model for early development and diseases, thus enabling teratogenicity testing in a cell culture system and enabling the generation of disease-specific cell lines [6]. Human ESC are generally generated form blastocysts produced by *in vitro* manipulations such as IVF and ICSI; however, *in vitro* manipulated embryos may already possess abnormalities that can be maintained in the ESCs lines generated from these embryos. On the other hand, if ESCs reflects the abnormalities of the embryo, they could be used as a method of testing quality of the embryos produced by assisted reproduction techniques. Moreover, these ESCs could enable us to analyze possible causes of the anomalies observed in adults generated by assisted reproductive technologies without the need to generate new animals.

Recently, it has been reported that ICSI procedures produce primary epimutations in mice that are, nevertheless, corrected in the germ line by epigenetic reprogramming and thus not propagated to subsequent generations [7]. However, ICSI using DFS can cause secondary epimutations or affect metastable epialleles and these abnormalities are transmitted to following generations. Many of these epialleles are comprised of transposable elements, and half of each mammalian genome is made up of these mobile, repetitive elements [8]. In the present study, we examined the mouse metastable epiallele *Axin 1* fused (*Axin1^Fu^*). This epiallele has a well-characterized locus, whose methylation pattern determines dramatic phenotypic outcomes. The *Axin1^Fu^* allele seems to be particularly vulnerable to environmental factors and its modifications may persist across several generations [9] through a process known as transgenerational epigenetic inheritance. *Axin1^Fu^* is a dominant gain-of-function allele that has a 5.1-kb intracisternal-A particle (IAP) retrotransposon (subtype I1) inserted in an antisense direction in intron 6 of *Axin1* [10]. The *Axin 1* gene regulates embryonic axis formation in vertebrates by inhibiting the Wnt signaling pathway [11]. The characteristic *Axin1^Fu^* phenotype consists of kinks in the tail caused by axial duplications during embryogenesis [11]. This phenotype is variably expressed among *Axin1^Fu^* individuals, and this variable expressivity correlates with differential DNA methylation at a cryptic promoter within the long terminal repeat (LTR) sequence of the IAP inserted in intron 6 of *Axin1* [12]. Rakyan *et al.* [12] observed that the methylation state of *Axin1^Fu^* in mature sperm reflects the methylation state of the allele in the somatic tissue of the animal, suggesting that it is not epigenetically reprogrammed during gametogenesis. In prior studies, we detected that ICSI causes epigenetic defects in preimplantation mouse embryos [13], [14], [15]. However, we are unaware if some of these epigenetic effects of ICSI will persist in subsequent generations when mice produced by ICSI are naturally mated.

This study was designed to determine whether the epigenetically inherited *Axin1^Fu^* allele is also sensitive to preimplantation development alterations induced by DFS-ICSI by examining whether DFS-ICSI causes a shift in *Axin1^Fu^* epiallele expression in the resulting progeny that is inherited by the next generation. In addition, we assessed the fertility of male mice generated by DFS-ICSI and determined whether ESC derived from blastocysts generated by DFS-ICSI differed from ESC derived from *in vivo* produced blastocysts as a possible method of testing assisted reproduction techniques without the need to generate new animals.

## Materials and Methods

### Ethics Statement

All experimental procedures using mice were approved by our Institutional Review Board (INIA), permit number CEEA2012/021, and performed according to the *Guide for Care and Use of Laboratory Animals* endorsed by the *Society for the Study of Reproduction* and European legislation.

### Animals and Embryo Production

Mice were fed a standard diet (Harland Ibérica) *ad libitum* and kept in a temperature- and light controlled room (22–24°C, 14L:10D). B6D2F1 (C57BL/6×DBA/2) female mice (8–10 weeks old) were superovulated by intraperitoneal injection of 7.5 IU of equine chorionic gonadotropin (eCG; Foligon 5000 Intervet), followed 48 h later by 7.5 IU of human chorionic gonadotropin (hCG; Veterin Corion, Equinvest) [16]. Oocytes in the superovulated B6D2F1 females were obtained from the ampulla of the oviduct and fertilized by ICSI using the DFS of B6D2F1 males [5] to generate ICSI embryos and then transferred at 2-cell stage to generate animals to study fertility. Embryos produced by B6D2F1 females naturally mated with B6D2F1 males and *in vitro* cultured from zygote to 2-cell, were used as controls. In the experiment to analyze postnatal expression of an epigenetically sensitive allele, oocytes obtained from B6D2F1 superovulated females were fertilized with the sperm of 129/Rr *Axin1^Fu^* ^/+^ males to generate an ICSI *Axin1^Fu^* group [9]. Animals produced by natural mating of B6D2F1 females with 129/Rr *Axin1^Fu/+^* males formed the *Axin1^Fu^* control *in vivo* group. In addition, a 2-cell embryo transfer *Axin1^Fu^* group was set up using zygotes obtained 0.5 day p.c. from the uterus of superovulated B6D2F1 females naturally mated with 129/Rr *Axin1^Fu^* ^/+^ males. All embryos obtained were cultured in KSOMaa+BSA for 24 h and those reaching the 2-cell stage were transferred to CD1 pseudopregnant females [17]. The tail phenotypes of the ICSI *Axin1^Fu^* group, 2-cell embryo transfer *Axin1^Fu^* group, and natural mating offspring group were analyzed and genotyped [9] to establish kinkiness categories. The tail phenotypes of the obtained pups were classified as no visible kink or slight kinking (1) (a small kink forming an angle <45° to the main tail axis), medium kinking (2) (one kink >30° but <45°), and kinky or very kinky (3) (several kinks >45°). The pups were genotyped by multiplex PCR of genomic DNA [12].

### ICSI using Frozen-Thawed Sperm

ICSI was performed as previously described [18]. Epididymal sperm cells collected in a minimal volume for freezing-thawing were placed in the bottom of a 1.5-ml polypropylene centrifuge tube and overlaid with the volume of fresh medium necessary to obtain a final concentration of 2.5 million cells per ml. The sperm extender used did not contain cryoprotectants such as EDTA or EGTA to induce DNA fragmentation [2]. Sperm samples were frozen in liquid nitrogen and stored for periods ranging from 1 day to 4 weeks at −80°C. Asepsis was maintained throughout the procedure. A volume of frozen-thawed sperm cells was mixed with 5 volumes of a 10% solution of polyvinyl-pyrrolidone (PVP; Mw 360,000) in M2 to give a final volume of 40–50 µl and placed in a culture dish for microinjection. ICSI was performed in M2 medium at room temperature. Sperm were mixed with M2 medium containing 10% PVP to reduce stickiness. Individual sperm heads, either mechanically obtained by decapitation using the piezo unit (for fresh sperm) or by freezing/thawing, were injected into oocytes as groups of ten oocytes. After a 15 min recovery period at room temperature in M2 medium, surviving oocytes were returned to mineral oil-covered KSOM and cultured at 37°C in a 5% CO2 air atmosphere for up to 24 h. Embryos that reached the 2-cell stage were transferred to the oviduct of Day 0.5 pseudopregnant females.

### Male Fertility Tests

Three virgin female B6D2F1 mice of 8–12 weeks of age were partnered with each male produced by ICSI or by natural mating on 5 consecutive days. The males in each group were classified according to age as young (4–6 months), adult (10–12 months) or old (16–18 months age) (control group: N = 16, N = 10 and N = 14; ICSI group: N = 16, N = 23, and N = 14 respectively). Every day during cohabitation, females were examined for plugs as evidence of mating. On gestation Day 14, females were euthanized using CO_2_ and the variables percentage of pregnant females, number of vaginal plugs, resorptions per litter and litter size recorded. Live fetuses were euthanized after examination. This fertility study was repeated 2–3 times.

### Sperm Motility

Adult 9-month-old ICSI males were sacrificed by cervical dislocation. The testis, epididymis, and vas deferens were immediately removed, and fat and veins dissected away to avoid contamination. For the motility test, the sperm were harvested into a 35 mm-well containing 500 µl of M2 medium (Sigma-Aldrich) by exerting soft pressure from the cauda epididymis to the end of the vas deferens with the help of watchmaker’s tweezers. The sperm sample was incubated at 37°C for 15 min until the sperm were homogeneously distributed in the M2 drop. A sample of 25 µl from the surface of the drop (swim-up) was placed on a microscope slide to obtain quantitative sperm motility variables. Sperm motility and progressive motility measurements were analyzed using an Integrated Semen Analysis System (ISAS). The parameters used for this analysis were SPV (Smoothed Path Velocity), TV (Track Velocity), STR (Straightness: ratio of VSL/VAP) and ALH (Amplitude of Lateral Head displacement), based on total motility, progressive motility and speed (static, medium and slow sperm cells) [19]. For sperm counts, a sample of sperm was diluted 1/10 in milli-Q water and 10 µl were placed in a Bürker chamber to obtain sperm cells concentrations (million spermatozoa/ml) using a standard procedure.

### Histological and TUNEL Assessment of the Testes

Both testes were fixed in Bouin’s solution for 24 h. The immersion-fixed testes were processed for paraffin embedding and posterior sectioning. Sections (5-µm thick) across the seminiferous tubules were deparaffinated, hydrated and stained with hematoxylin for histological examination. The TUNEL assay for apoptotic cell detection was performed using the *In Situ* Cell Death Detection Kit (Boehringer Mannheim GmbH, Mannheim, Germany) according to the manufacturer’s instructions. Apoptosis was visualized using anti-fluorescein antibody Fab fragments conjugated with alkaline phosphatase (AP) and converter-AP. The number of TUNEL positive cells in approximately 250 seminiferous tubules of each mouse was counted, and apoptotic indices then determined by calculating the ratio of the total number of TUNEL positive cells/number of counted seminiferous tubules. For histological examination, seminiferous tubule cross sections were randomly chosen in three non-serial sections per animal, totaling more than 100 tubules/animal and the percentage of tubules showing abnormal spermatogenesis (irregularly outlined seminiferous tubules showing disarranged cell layers and loss of germ cells, or premature release of germ cells into the seminiferous tubule lumen, or an empty tubular lumen) and abnormal tubule morphology (empty tubules and seminiferous tubules containing only Sertoli cells) compared in the control (N = 5) and ICSI (N = 8) groups. Since germ cell numbers vary in tubule sections between stages I-VIII and stages IX-XIV, the same number of tubules at each of these two stages was considered per animal.

### Embryonic Stem Cell Production and Karyotyping

Female mice (8–10 weeks old) were superovulated as described above [16], and *in vivo*-produced blastocysts were collected 3.5 days after the hCG injection and used as controls. ICSI-produced blastocysts were obtained as described above. The *in vivo*- and ICSI-produced blastocysts were plated individually onto 96-well plates containing mitomycin-C treated (Sigma-Aldrich corporation St. Louis, MO, USA) mouse embryonic fibroblast (MEF) cells on 0.1% gelatin-coated tissue plates containing Dulbecco’s modified Eagle medium (DMEM plus 4500 mg/l glucose, glutaMAX, and pyruvate; Invitrogen, Carlsbad, CA, USA) supplemented with 20% FBS (PAA Laboratories Cölbe Germany), 2 mM glutamine, 1 mM MEM nonessential amino acids solution, 1 mM β-mercaptoethanol, 1000 U/ml LIF, an antibiotic mixture containing 100 U/ml penicillin and 100 µg/ml streptomycin, 3 µM GSK3Beta inhibitor (Stemolecule CHIR99021, Stemgent, San Diego, CA, USA) and 0.5 µM MEK inhibitor (Stemolecule PD0325901, Stemgent, San Diego, CA, USA). Blastocysts were allowed to attach to supportive MEFs and to expand for four days. After this, all cell clumps were disaggregated by incubation in 0.05% Trypsin/0.02% EDTA in Ca^2+^-free and Mg^2+^-free Dulbecco’s phosphate-buffered saline (PBS) at 37°C for 3 min and transferred into 96-well plates containing MEFs and ES medium. Approximately 4 days after trypsinization, compact ESC colonies could be detected and these were then trypsinized into 24- well plates containing MEFs and ES medium lacking GSK3Beta and MEK inhibitors. For cell line expansion, cells were trypsinized at 80% confluence, and clones not reaching confluence plated onto the same plate size. When ESCs were transferred to a 35-mm dish, this was considered the first passage. The culture medium was changed daily.

For karyotyping, ESC were arrested in metaphase by supplementing the culture medium with 0.1 µg/ml Karyomax Colcemid Solution (Gibco, Paisley, Scotland, UK) for 2 h at 37°C in a 5% CO2 air atmosphere. Cells were then disaggregated by incubation in 0.05% Trypsin/0.02% EDTA in Ca^2+^-free and Mg^2+^-free PBS at 37°C for 2 min. After pipetting, a single cell suspension was washed twice in PBS by centrifugation at 200 G for 5 min. The pellet obtained was subjected to hypotonic shock by resuspending in 0.075 M KCl for 15 min at 37°C. After a second centrifugation step, the hypotonic solution was removed and the pellet was fixed in a methanol/acetic acid solution (3∶1; vol/vol) by gently pipetting. Ten minutes later, cells were re-pelleted and fixed for a second time. Before slide mounting, cells were washed twice with PBS. The slides were dried overnight at 55°C, stained in freshly made 10% Giemsa solution for 30 min, and rinsed with distilled water. Finally, chromosome spreads were observed using an Optishot II microscope (Nikon, Tokyo, Japan) at a magnification of 1000x [20].

### RNA Isolation, cDNA Synthesis, and qPCR

Poly (A) RNA was extracted from 7 ICSI-derived and 7 *in vivo*-derived ESC lines at passage 0 and passage 10 using the Dynabeads mRNA Purification Kit (Life Technologies, Oslo, Norway) following the manufacturer’s instructions with minor modifications. Briefly, 100 µl of lysis buffer were added to the sample and incubated at RT for 10 min with gently shaking. Then, 20 µl of beads were added and incubated at RT for 5 min with gentle shaking, allowing beads/mRNA complexes formation. Finally, beads/mRNA complexes were washed twice in washing buffer A and twice in washing buffer B, and resuspended in 10 mM Tris-HCl pH 7.5. Immediately after extraction, the RT reaction was carried out following the manufacturer's instructions to produce cDNA. Tubes were heated to 70°C for 5 min to denature the secondary RNA structure, allowing Random Primer and Oligo dT annealing, and the RT mix was then completed with the addition of 0.375 mM dNTPs (Biotools, Madrid, Spain), 6.25 U RNAsin RNAse inhibitor (Promega, Madison, WI, USA), MMLV HP RT 10X reaction buffer, 5 mM DTT and 5 U MMLV high performance reverse transcriptase (Epicentre, Madison, WI, USA). Tubes were first incubated at room temperature for 10 min and then at 42°C for 60 min to allow the reverse transcription of RNA, followed by 70°C for 10 min to denature the RT enzyme. To detect each transcript, we used 2 µl of the cDNA sample in the RT-PCR. mRNA transcripts were quantified by real-time qRT-PCR [21]. Two replicate PCR experiments were conducted for all genes of interest. Experiments were designed to compare the relative levels of each transcript and those of glyceraldehyde-3-phosphate dehydrogenase (*Gapdh*) in each sample. PCR was performed by adding a 2-µl aliquot of each sample to the PCR mix (GoTaq qPCR Master Mix, Promega, Madison, WI, USA) containing the specific primers. Primer sequences are provided in Table S1. The comparative cycle threshold (CT) method was used to quantify expression levels [22]. Quantification was normalized to the endogenous control *Gapdh*. Fluorescence was acquired in each cycle to determine the threshold cycle, or the cycle during the log-linear phase of the reaction wherein fluorescence increased above background for each sample. Within this region of the amplification curve, a difference of one cycle is equivalent to doubling of the amplified PCR product. According to the comparative CT method, the CT value was determined by subtracting the *Gapdh* CT value for each sample from the CT value of each gene in the sample. CT was calculated using the highest sample CT value (i.e., the sample with the lowest target expression) as an arbitrary constant to be subtracted from all other CT sample values. Fold changes in the relative gene expression of the target were determined using the formula 2^–CT^.

### Statistical Analysis

Pregnancy rates, number of implantations per litter, numbers and percentages of live births, resorptions, and dead fetuses per litter were compared between the different groups by one-way ANOVA and mean comparisons made using Holm-Sidak post hoc tests. The effect of treatment on tail phenotype was analyzed by multinomial logistic regression (SigmaStat, Jandel Scientific, San Rafael, CA). The number of pups showing the penetrant or silent phenotype in each litter was entered in the statistical model as covariates. mRNA expression data were also analyzed by one-way repeated-measures ANOVA with arcsine data transformation when necessary. When main effects were detected, Holm-Sidak post hoc tests were used to make comparisons with the control group. All statistical tests were performed using the *SigmaStat* (Jandel Scientific, San Rafael, CA) package.

## Results

### ICSI using DNA-fragmented Sperm Reduces Embryonic Stem Cell Derivation Efficiency without Affecting the Karyotype

To examine the effect of DFS-ICSI on ESC derivation, 31 DFS-ICSI-derived blastocysts and 30 control *in vivo*-derived blastocysts were plated onto feeder layers of MEFs in the presence of GSK3β and MEK-inhibitors until the establishment of ESC lines. On Day 4 after seeding on MEFs, we observed the attachment and proliferation up to Day 7 of 10 DFS-ICSI-derived cell clumps and 27 *in vivo*-derived cell clumps, that were trypsinized and passed onto new MEF-coated plates to establish ESC lines. Three days after passaging, 7 DFS-ICSI-derived ESC lines and 25 *in vivo*-derived ESC lines were obtained indicating an efficiency of 83% for the *in vivo*-derived ESC lines and 23% for the DFS-ICSI-derived ESC lines. Both groups of ESC lines were then subject to chromosome analysis. No karyotype abnormalities were detected and appropriate chromosome numbers were observed in 81% (170/209 metaphase spreads) of metaphase spreads prepared from DFS-ICSI-derived ESC lines and 78% (117/150 metaphase spreads) from *in vivo*-derived ESC lines.

### Gene Expression in DFS-ICSI-Produced Embryonic Stem Cells

**Figure 1:**
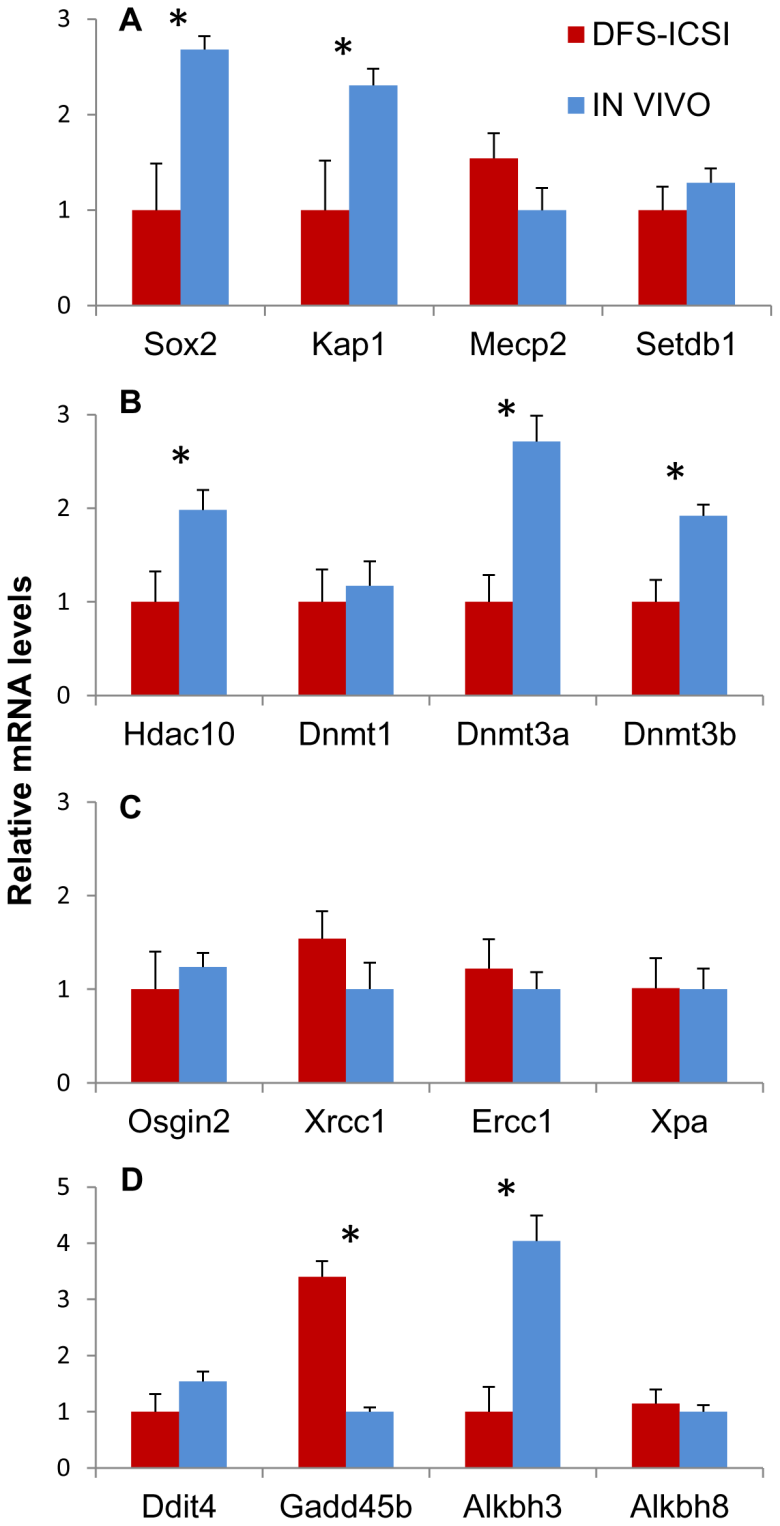
mRNA expression in DFS-ICSI- and *in vivo*-derived ESC lines at early passage (passage 0). (A) pluripotency and epigenetic repression genes; (B) DNA methylation and histone acetylation genes; (C) oxidative stress, base excision repair (BES) and nucleotide excision repair (NES) genes; and (D) DNA damage and repair genes. * indicates statistical differences for each gene transcript at P≤0.05; error bars represent SEM.

To compare the DFS-ICSI-derived and *in vivo*-derived ESC lines, gene expression profiles were examined upon early passage (passage 0) and after several passages (passage 10). The genes selected were *Sox2*, *Kap1*, *Mecp2* and *Setdb1* due to their known roles in pluripotency and epigenetic repression. The profiles obtained indicated that *Sox2* and *Kap1* were significantly downregulated in the early passage DFS-ICSI-derived ESC lines compared to the *in vivo*-derived ESC lines (Figure 1A). The expression of genes involved in DNA methylation and histone acetylation, *Hdac10*, *Dnmt3a* and *Dnmt3b*, was also significantly lower in the early passage DFS-ICSI-derived than the *in vivo*-derived ESC lines (Figure 1B). In contrast, no significant differences were detected between the two ESC groups in their expression patterns of *Osgin2*, *Ercc1*, *Xrcc1* and *Xpa* as markers of oxidative stress, base excision repair (BES) and nucleotide excision repair (NES) (Figure 1C). Finally, we assessed DNA damage and repair by analyzing *Ddit4*, *Gadd45b*, *Alkbh3* and *Alkbh8* expression. Significant upregulation was observed of *Gadd45b* and downregulation of *Alkbh3* in the DFS-ICSI-derived ESC lines upon early passage (Figure 1D). No differences were detected in the expression profiles of the late passage cell lines (Figure S1).

### Effect of ICSI using DNA-fragmented Sperm on the Sperm Count and Motility

No significant differences were recorded in the average weight of either testes or epididymis in the DFS-ICSI-produced and control male mice. However, four DFS-ICSI-produced males (20%) (N = 20 mice analyzed) showed a very low testes weight (<0.07 g vs 0.20 g in both WT and rest of ICSI mice), and morphological abnormalities (atrophied testes without presence of spermatozoa). In addition, in three of the DFS-ICSI animals (15%), testes weight was high (≥0.26 g; above the 90th percentile for the colony). In contrast, the control males all showed a normal testes weight and normal testicular morphology (N = 20). Significant differences (P = 0.002) were detected in the mean number of sperm collected from the cauda epididymis and deferent conduct (6.77±1.2×10^6^ spz/ml for N = 8 DFS-ICSI males versus 15.61±2.2×10^6^ spz/ml for N = 7 control males). No differences were observed between the DFS-ICSI and control groups in overall sperm motility (64.25% and 72.79% respectively) and progressive sperm motility (32.61% and 32.75% respectively).

### Effect of ICSI using DNA-fragmented Sperm on the Copulation and Fertility Rates of the Male Progeny

Pregnancy rates recorded in the B6D2F1 female mice partnered with DFS-ICSI-produced males were significantly reduced compared to the rates observed in females mated with control males (49.96% ±5.24, N = 237) vs. 86.52% ±3.13, 138), Table 1). Moreover, 100% of the females with vaginal plugs mated with control mice became pregnant yet pregnancy was only observed in 68.48% of the females with vaginal plugs mated with DFS-ICSI males. In DFS-ICSI males, a statistically significant decrease in pregnancy rates related to the age was observed. Thus, the ageing phenotype previously observed in these animals [5] could affect fertility as well. No differences were detected in litter size between the DFS-ICSI and control groups. However, significantly higher percentages of pregnant females with resorptions or mummified fetuses were recorded for the DFS-ICSI males. Furthermore, a statistically significant increase in the percentage of pregnant females with resorptions was observed in old males from the control group (Table 1). Some authors have described a decreased reproductive potential (in natural conception, *in vitro* blastocyst development, and implantation potential) during ageing [23]. Although old males from the control group showed high pregnancy rates, it could be possible that aged sperm that should not fertilize yields a high number of resorptions. On the contrary, this difference could not be found in old males from the DFS-ICSI group, as resorption rates were high at all ages. In addition, more than 15% (8/53) of the males in the DFS-ICSI group could be considered infertile since no pregnancies were recorded in response to their partnering with at least 6 virgin females. In a subsequent examination of the testes of these animals, we observed reduced testis sizes and low amounts of sperm.

**Table 1:** Results of matting of DFS-ICSI and control male mice at 4–6 months (young), 10–12 months (adult), and 16–18 months (old) of age.

Male group	Age (N)	No. ofFemales	No. vaginalplugs	Pregnantfemales (%)	Average of puppiesby litter	Total No. of resorptions(females %)
Control	Young (16)	45	43	43 (89,6±3,7)^a^	7,7	6 (13,8±5,2)^a^
	Adult (10)	48	38	38 (86,7±7,1)^a^	7,92	5 (12,9±6,5)^a^
	Old (14)	45	39	39 (83,3±5,3)^a^	6,6	14 (36,8±6.1)^b^
	Total (40)	138	120	120 (86,5±3,1)^a^	7,4	25 (21,2±3,7)^a^
DFS-ICSI	Young (16)	48	34	29 (60,4±9,7)^b^	8,17	12 (41,4±11,1)^bd^
	Adult (23)	138	83	65 (47,1±7,6)^c^	7,06	31 (47,7±8,7)^cd^
	Old (14)	51	29	19 (37,2±10,5)^d^	7,8	7 (36,8±12,6)^bd^
	Total (53)	237	165	113 (49,9±5,2)^c^	7,6	50 (36,2±5,9)^bd^

Data are mean ± SEM. Within rows, values followed by different superscript letters differ significantly (P≤0.05).

### Histology and TUNEL Labeling of Testicular Tissue Sections

**Figure 2:**
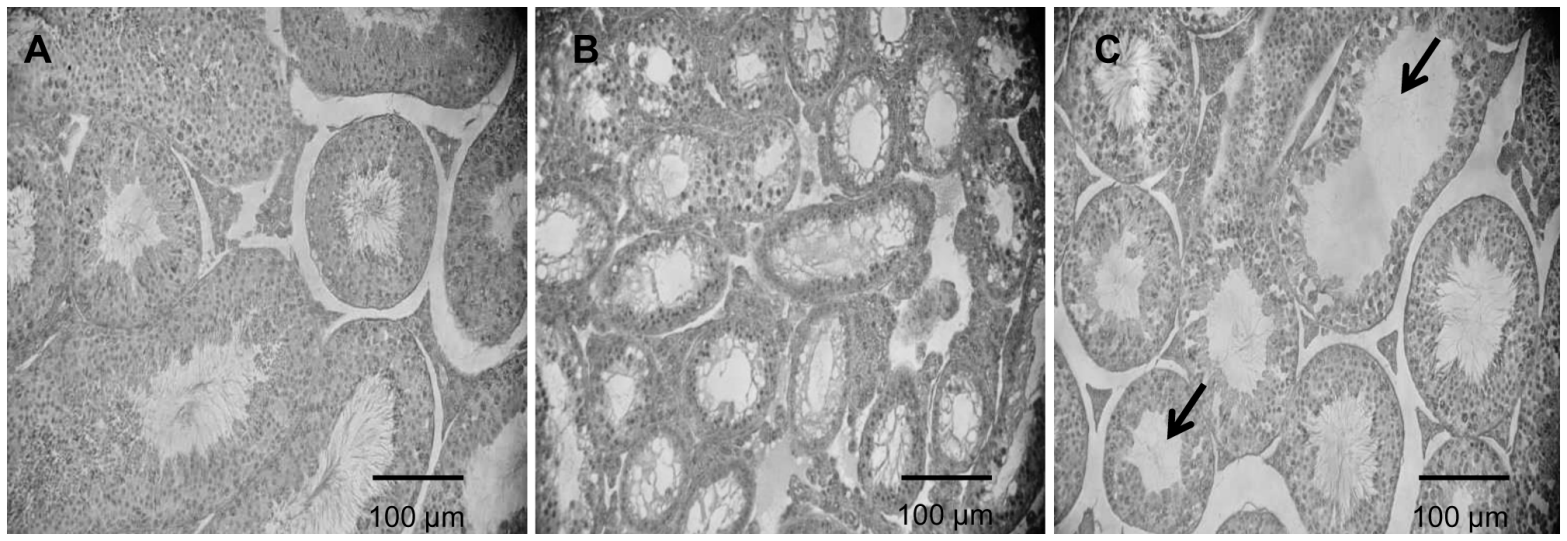
Histological comparisons between seminiferous tubules in adult offspring produced by DFS-ICSI or natural mating. (A) Seminiferous tubules in control mice show a normal shape with germ cells organized in concentric layers and exhibit ongoing germ cell production. (B) Abnormal seminiferous tubules observed in the testes of infertile DFS-ICSI-produced males (20% of the DFS-ICSI male mice with small testes); note their irregular shape and loss of germ cells in many atrophied seminiferous tubules. (C) The testis of an DFS-ICSI male showing reduced fertility (80% of DFS-ICSI males) containing both tubules showing a normal appearance and ongoing spermatogenesis as well as severely degenerated tubules, which are either empty or have only a small germ cell population (arrows).

In hematoxylin-stained cross-sections of the seminiferous tubules of adult mice produced by DFS-ICSI, we observed that the mice with small testes (approximately 20%) had evident signs of abnormal spermatogenesis and abnormal tubule morphology. In addition, we noted a lack of germ cells in the atrophied tubules indicating subfertility or infertility (Figure 2B). When we examined 614 control tubule cross-sections (N = 8), it was noted that 24.1±3.2% of the sections showed reduced spermatogenesis and an abnormal tubule morphology (Figure 2A). In contrast, out of 1682 tubule cross-sections examined in the DFS-ICSI group (N = 8), reduced spermatogenesis was observed in 43.3±3.1% and an abnormal morphology in 14.4±3.9% (P = 0.002) (Figure 2C).

The TUNEL assay was performed on paraffin-embedded cross-sections of testicular tissue obtained from DFS-ICSI (N = 7) (Figure S2C, D) and control (N = 5) (Figure S2A, B) adult male mice. At least 200 seminiferous tubules were examined per animal. Our results indicate that compared to control mice, the testes of DFS-ICSI males showed more TUNEL-positive cells/cross-sectioned tubule (apoptosis index) and more spermatogenic cells undergoing apoptosis (Figure S2E).

### ICSI using DNA-fragmented Sperm Affects the Postnatal Expression of an Epigenetically Labile Allele, *Axin1^Fu^*

**Figure 3:**
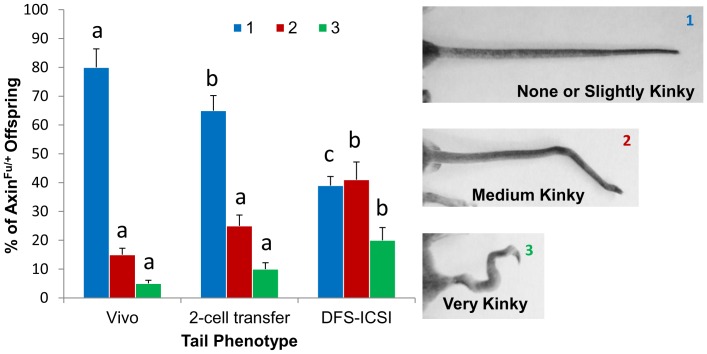
Effect of DFS-ICSI on the tail kinking phenotype of the offspring. Tail phenotypes (1: none or slightly kinky; 2: medium kinky; 3: very kinky) recorded in all the *Axin1^F^*/+ offspring in the groups: *in vivo*-produced controls, oocytes fertilized *in vivo*, cultured for 24 h and transferred at the 2-cell stage, and DFS-ICSI-fertilized oocytes transferred at the 2-cell stage. DFS-ICSI led to a kinkier tail phenotype. Error bars represent mean ± SD. Bars with different lowercase letters (a, b, c) represent significant differences for each phenotype (P≤0.01).

The *Axin1^Fu^* ^/+^ progeny of oocytes fertilized by DFS-ICSI (N = 90) were more likely to have a kinky tail compared to either of the two control groups (*Axin1^Fu^* ^/+^2-cell transfer group (N = 100) and natural mating group (N = 125) (P<0.01, Figure 3). Both control groups also differed (P<0.05) according to their no kinks or slightly kinky tail phenotype distributions (Figure 3). Our results indicate that the DFS-ICSI fertilization of oocytes led to the birth of more pups that expressed an active *Axin1^Fu^* ^/+^ epiallele, resulting in more pups with a kinky tail. This could not be attributed to the different survival of embryos of a given genotype, nor was it the consequence of superovulation, culture for 24 h until the 2-cell embryo stage or embryo transfer to a pseudopregnant recipient dam.

## Discussion

In our model of ICSI we used frozen-thawed sperm because we have previously observed that freezing in the absence of a cryoprotectant gives rise to DNA-fragmented spermatozoa (DFS) with double-strand DNA breaks [5]. In effect, when ICSI is used in human assisted reproduction to overcome male infertility, sperm with damaged DNA incapable of oocyte fertilization *in vivo* are used for fertilization *in vitro*. ICSI with DNA fragmented sperm produces genetic and epigenetic alterations in preimplantation embryos that affect the phenotype of the offspring, and lead to the long term manifestation of a variety of deleterious phenotypes in mice [5]. Here, we used ESCs derived from embryos produced by DFS-ICSI to examine the effects of DFS-ICSI without the need to produce animals. Our data indicate that DFS-ICSI-produced embryos show a reduced potential to generate ESC lines compared to *in vivo*-produced embryos, and that during early passages these ESCs differ in their expression of certain genes. Once passaged 10 times, however, such gene expression differences were lost, confirming the idea that mouse ESCs of different origins will eventually adopt a similar gene and protein expression profiles after several passages [24], [25]. The reduced derivation efficiency of ESC lines produced by DFS-ICSI could be a consequence of the low quality of these embryos both in terms of genetics and epigenetics, confirming the low rates of successful implantation and fetal development observed for these embryos [18]. Several studies have tried to correlate sperm DNA integrity with embryo quality and long term effects. Thus, 40% of embryos generated by ICSI using DFS showed abnormal chromosome segregation and chromosome fragmentation; and half of these embryos with abnormal chromosomes developed into normal-looking blastocysts and were capable of implantation. However, almost all of them aborted spontaneously before embryonic Day 7.5 [26]. The reduced number of ESC lines derived here from our DFS-ICSI embryos and the fact that all the lines showed a normal karyotype suggests that embryos with an abnormal karyotype are unable to produce ESC lines.

Our observation of gene expression differences between early passaged DFS-ICSI-derived and *in vivo*-produced ESC lines including the down-regulation of both *Hdac10* and the *de novo* DNA methyltransferases *Dnmt3a* and *Dnmt3b* in the DFS-ICSI-derived ESC lines may confirm epigenetic differences among DFS-ICSI-generated embryos [5]. Hypomethylation in ESCs has been also linked to the down-regulation of *Dnmt3a* and *Dnmt3b* and to differentiation dissimilarities [27]. Moreover, the lower expression of *Sox2* observed here could be related to the reduced pluripotency of these ESCs. Also, the transcriptional repression of *Kap1* along with *Sox2*, *Hdac10*, *Dnmt3a*, and *Dnmt3b*, as modifiers of epigenetic gene silencing through the transcription of specific genes, involves changes in chromatin state. Interestingly, these genes are retained in the small fraction of sperm DNA bound by nucleo-histones [28], [29] suggesting that the mechanism whereby DFS-ICSI modifies phenotype could be related to mechanisms of epigenetic gene silencing. The higher expression of *Gadd45* in our DFS-ICSI-derived ESC lines may support some sort of DNA damage in these cells. In addition, *Gadd45* family proteins have been attributed a role in senescence and aging, and this phenotype is typical of DFS-ICSI-generated mice [5]. The reduced expression of the repair enzyme-coding *Alkbh3* [30] detected in our DFS-ICSI-derived ESC lines is in agreement with the premature aging of DFS-ICSI-generated mice [5].

When we examined the effects of DFS-ICSI on male fertility, we identified a group of mice showing small testes and infertility that represented 20% of the male animals produced. Remaining DFS-ICSI males with normal-sized testes showed reduced average fertility during all stages of life. Other studies have analyzed the effect of ICSI with normal sperm on fertility. When gene expression in the testes of both ICSI-produced and naturally conceived mice by micro-array analysis was examined, 474 (150 up-regulated and 324 down-regulated in ICSI mice) differentially expressed genes were identified representing several functional pathways, including those implicated in spermatogenesis, male meiosis I, spermatid development, gonad development and male genitalia development. It has been observed that such differential gene expression patterns are transmitted to the next generation [31]. It has been also reported that ICSI-derived mice exhibit a high level of spermatogenic cell apoptosis, suggesting a risk of the compromised fertility of male progeny [32]. Our observation of the reduced *in vivo* fertility of DFS-ICSI male mice is consistent with such findings and indicates that DFS-ICSI could compromise the fertility of the male offspring in other mammals.

In this study, we also examined the effects of DFS-ICSI on the postnatal expression of an epigenetically labile allele, *Axin1^Fu^*. Our results indicate that DFS-ICSI perturbs the epigenetic reprogramming of *Axin1^Fu^* causing a shift towards the active state of the epiallele. The manifestation of this was the birth of more pups expressing the active epiallele rendering a kinky tail phenotype. Similar results have been reported for the effect of 4 days of IVC on the *Axin1^Fu^* and *A^vy^* alleles [33]. Collectively, our results suggest that such effects of ICSI and IVC are likely to affect metastable epialleles in general and reveal that while primary epimutations produced by ICSI in mice can be properly corrected in the germ line by epigenetic reprogramming [7], the alterations produced by ICSI in some metastable epialleles like *Axin1^Fu^* are propagated to subsequent generations. With regard to the question of whether the rest of the genome may have mechanisms similar to the regulation of the *Axin1^F u^*allele, we know that transposable elements represent up to 45–50% of mouse and human genomes [8]. Many of these new alleles produced by the insertion of a transposable element are expressed during preimplantation development [34]. Given the conservation of epigenetic mechanisms during evolution in mice and humans, it is likely that similar mechanisms of metastable epialleles will be active in humans. Our results point to the notion that changes in the epigenetic state of the genome can be induced early in development by environmental conditions, and that these changes can have consequences for both gene expression in adulthood [35], [36] and the inheritance of epigenetic phenotypes. We are unaware of the reason why DFS-ICSI renders a kinkier tail phenotype, though a kinked tail has been described as an embryopathy produced by oxidative DNA damage due to ROS [37]. One of the targets for oxidative DNA damage is the methylated base m^5^C found in mammalian DNA. Thus, if DFS-ICSI increases ROS levels in the embryo, this may produce oxidative DNA damage and preferentially affect m^5^C.

In humans, ICSI is currently used as a successful infertility treatment. Although a significantly increased risk of birth defects in infants conceived by assisted reproductive technology (ART) has been described in the last years, it has been reported that there is no risk difference between children conceived by IVF and/or ICSI [38]. Some studies have suggested that this increased risk may be due to the underlying infertility of the couples pursuing ART, and not to ARTs themselves [38], [39], [40]. Furthermore, significant limitations of human studies are the lack of a good comparison group for IVF or ICSI, which would be babies naturally conceived by infertile couples rather than babies conceived by overall population, and the low power of the studies due to the rarity of the diseases [39]. However, a potential risk of ICSI is the use of spermatozoa with apparently normal morphology but with DNA fragmentation. A significant proportion of infertile men have elevated levels of DNA damage in their ejaculated spermatozoa [41]. Sperm DNA damage is a useful biomarker for male infertility diagnosis and it is associated with reduced fertilization rates, embryo quality and pregnancy rates, and higher rates of spontaneous miscarriage and childhood diseases [42]. It remains unclear whether assisted reproductive techniques can compensate for DNA damage. Hence, studies conducted with animal models are particularly important. Some of the effects described in our manuscript regarding male infertility in DFS-ICSI mice have been previously described in mice produced by ICSI with intact fresh sperm. Yu *et al.* reported decreased testis weight, abnormal tubule morphology and increased apoptosis in testis of adult mice produced by ICSI with fresh sperm [32]. Other studies with ICSI using fresh sperm described diverse alterations as transcriptome perturbations that remained at the neonatal stage [43], or alterations in glucose parameters in adult mice [44]. Studies that induce sperm DNA damage and evaluate its biologic effects on the offspring and on next generations are of vital importance. To date, few animal studies have assessed the effects of induced sperm DNA damage on fertilization and embryo development. Yamagata *et al.* described abnormal chromosome segregation in embryos generated by ICSI with fresh sperm or with DFS. We have previously described that more severe abnormalities appear when ICSI is performed with DFS compared to ICSI performed with fresh sperm. Although there were no differences in fertilization and embryo developmental competence, pregnancy rates, live offspring rates and survival after 25 weeks were significantly lower. Furthermore, ageing phenotype and tumor development were observed in DFS-ICSI animals but not in animals generated by ICSI with fresh sperm [5]. Thus, from these data we could speculate that although ICSI *per se* (performed with fresh sperm) can produce several alterations, abnormal phenotypes are more severe when ICSI is performed with DFS. However, comparative analyses of ICSI with fresh sperm *vs.* DFS-ICSI are very important.

Our findings offer new motives for current concerns over the safety of ICSI with DFS. Especially worrying is the frequent use of ICSI in cases of severe male factor infertility, since a significant proportion of the spermatozoa used for ICSI are likely to have fragmented DNA. The subacute nature of some of the aberrant embryo modifications induced by ICSI with DFS means that many of these changes will be undetected in the short term. Moreover, embryo development to the blastocyst stage, considered a hallmark of ART system efficiency, is often possible despite detrimental environmental effects and longer term consequences of this procedure [5]. Our first conclusion is that we should only use early passage DFS-ICSI-derived ESC lines to assess specific alterations associated with the DFS-ICSI technique. Secondly, we demonstrate here that DFS-ICSI in the mouse modifies reprogramming in a manner that favors the active state of the *Axin1^Fu^* epiallele, and that this epigenetic alteration is transmitted to the following generations. Thirdly, our findings also reveal a dramatic influence of DFS-ICSI on the reproductive lifespan of male progeny. This could have significant implications for the reproductive management of livestock, endangered species and also humans.

## Supporting Information

Figure S1 **mRNA expression in DFS-ICSI- and** ***in vivo*** **-derived ESC lines at late passage (passage 10).** * indicates statistical differences in gene transcription at P<0.05; error bars represent SEM.
(TIF)

Figure S2 **TUNEL analysis in testes from DFS-ICSI- and** ***in vivo*** **-produced mice.** TUNEL labeling of a cross-section of testes from *in vivo-* (A,B) and DFS-ICSI-produced (C,D) mice. (E) The mean number of TUNEL-positive cells/tubule cross section was higher in the testes of DFS-ICSI-produced mice than control testes. *P<0.05; error bars represent SEM.
(TIF)

Table S1 Primers used for RT-PCR.
(DOCX)
