# Supplementary figures and images for: Intracytoplasmic Sperm Injection Using DNA-Fragmented Sperm in Mice Negatively Affects Embryo-Derived Embryonic Stem Cells, Reduces the Fertility of Male Offspring and Induces Heritable Changes in Epialleles
Source: PLoS One. 2014 Apr 17;9(4):e95625. doi: 10.1371/journal.pone.0095625 (PMC3990723; doi:10.1371/journal.pone.0095625)

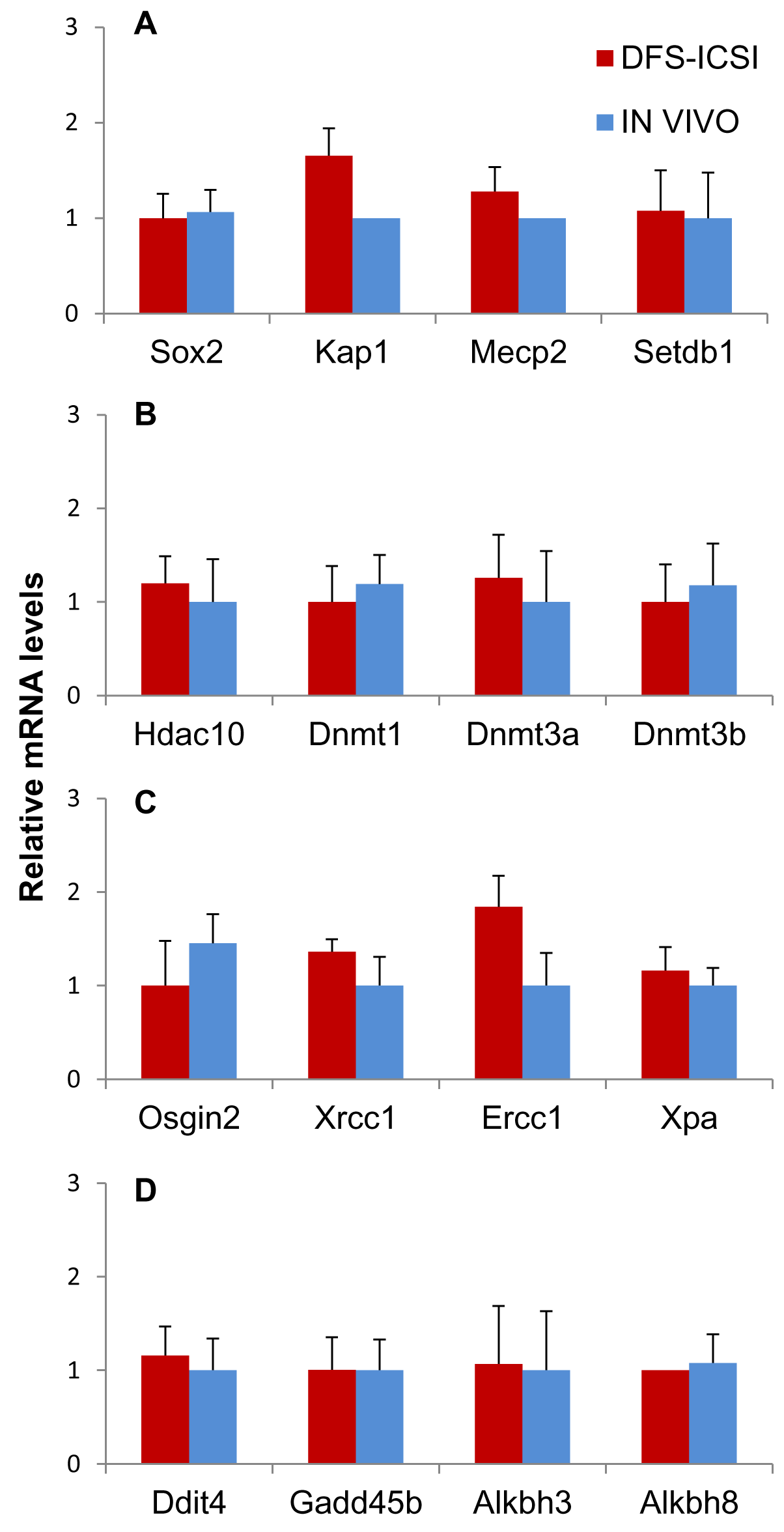

Supplement: Figure S1 — mRNA expression in DFS-ICSI- and in vivo -derived ESC lines at late passage (passage 10). * indicates statistical differences in gene transcription at P<0.05; error bars represent SEM. (TIF) [file pone.0095625.s001.tif]

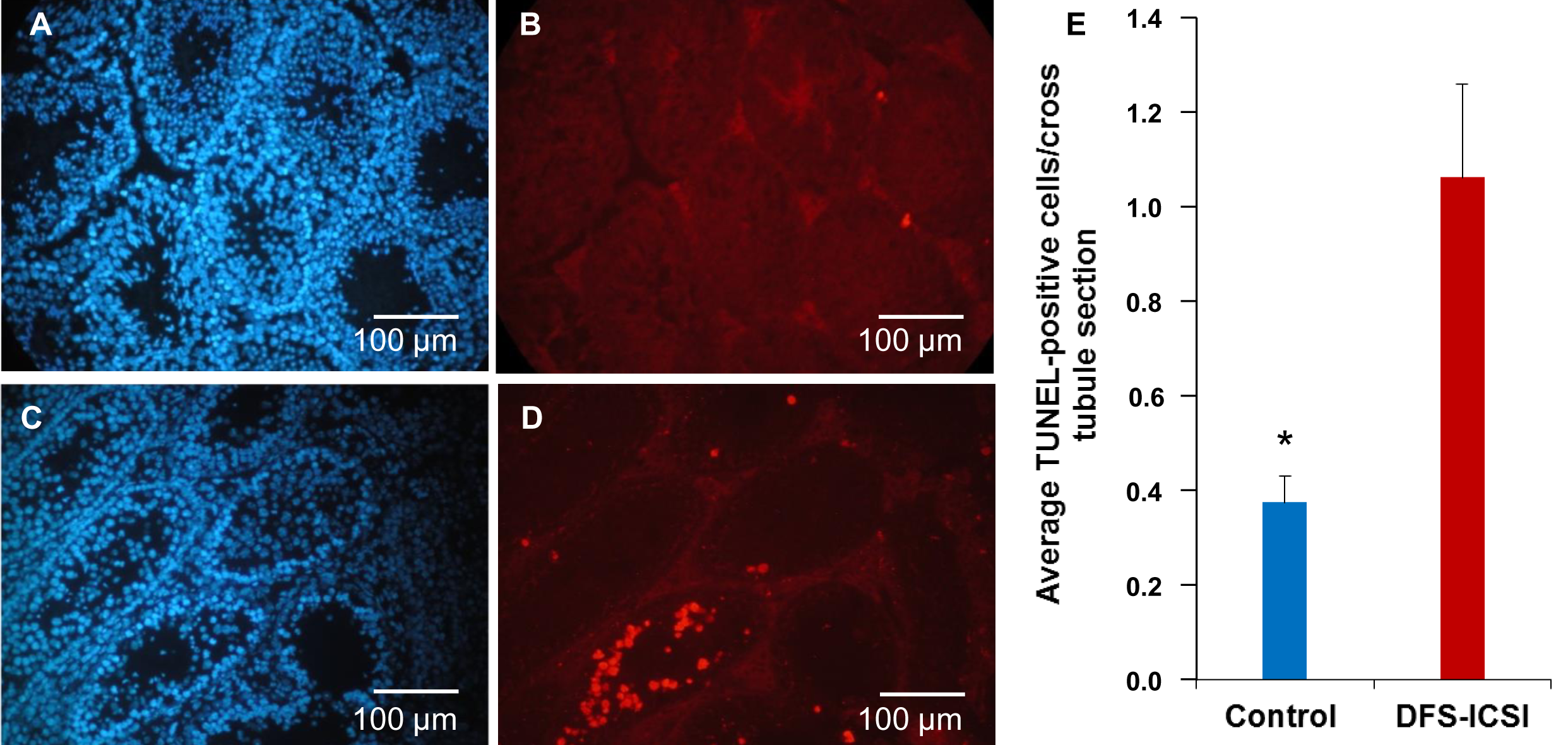

Supplement: Figure S2 — TUNEL analysis in testes from DFS-ICSI- and in vivo -produced mice. TUNEL labeling of a cross-section of testes from in vivo- (A,B) and DFS-ICSI-produced (C,D) mice. (E) The mean number of TUNEL-positive cells/tubule cross section was higher in the testes of DFS-ICSI-produced mice than control testes. *P<0.05; error bars represent SEM. (TIF) [file pone.0095625.s002.tif]
