# Supplementary material for: Intracytoplasmic Sperm Injection Using DNA-Fragmented Sperm in Mice Negatively Affects Embryo-Derived Embryonic Stem Cells, Reduces the Fertility of Male Offspring and Induces Heritable Changes in Epialleles
Source: PLoS One. 2014 Apr 17;9(4):e95625. doi: 10.1371/journal.pone.0095625 (PMC3990723; doi:10.1371/journal.pone.0095625)
Supplement: Table S1 — Primers used for RT-PCR. (DOCX) [file pone.0095625.s003.docx]

**Table S1.** Primers used for RT-PCR

| **Gene** | **Primer sequences 5´-> 3´** |
| --- | --- |
| ***H2az*** | AGGACGACTAGCCATGGACGTGTG / CCACCACCAGCAATTGTAGCCTTG |
| ***Sox2*** | GCACATGAACGGCTGGAGCAACG / TGCTGCGAGTAGGACATGCTGTAGG |
| ***Kap1*** | GGAATGGTTGTTCATTGGTG / ACCTTGGCCCATTTATTGATAAAG |
| ***Mecp2*** | ATATTTGATCAATCCCCAGGG / CTTAGGTGGTTTCTGCTCTC |
| ***Setdb1*** | CTTCTGGCTCTGACGGTGAT / GGAAGCCATGTTGGTTGATT |
| ***Hdac10*** | GTGCCTGCTTAGGAGCTCTG / CCTCCACCCTACAGAATTGG |
| ***Dnmt1*** | GCTTCTACTTCCTCGAGGCCTA / GTTGCAGTCCTCTGTGAACACTGTG |
| ***Dnmt3a*** | CACAGAAGCATATCCAGGAGTG / GTCCTCACTTTGCTGAACTTGGC  (/ |
| ***Dnmt3b*** | ACGTCAATCCTGCCCGCAAAGGT / ACTGGGTTACATGCCAGGAATCTT |
| ***Osgin2*** | TCCGGCCTTACTGCCGCTGA / TGGCTGGCTTGAGTTACGGCC |
| ***Xrcc1*** | AGAATGGCGAGGACCCGTAT / CTCTGGGATTGGCAGGTCAG |
| ***Ercc1*** | GTGCTGCTGGTTCAAGTGGA / GCAGTCAGCCAAGATGCACAT |
| ***Xpa*** | AATGCGGGAAAGAGTTCATGG / CATCAGCATCTCTGCAGCTGT |
| ***Ddit4*** | CTCTTGTCCGCAATCTTCGCT / GGACACCCCATCCAGGTATGA |
| ***Gadd45b*** | CTTCTGGTCGCACGGGAAGG / GCTCCACCGCGGCAGTCACC |
| ***Alkbh3*** | GTGGACTGGCACAGCGACGA / CCAGTCGGCTTGTGTGGCTCC |
| ***Alkbh8*** | AGGAAGGCCACACCTCCATCCC / CAGCCAGCGCATCGCAGACTA |
